# Signal Quality in Continuous Transcutaneous Bilirubinometry

**DOI:** 10.3390/s24186154

**Published:** 2024-09-23

**Authors:** Fernando Crivellaro, Anselmo Costa, Pedro Vieira

**Affiliations:** 1 Department of Physics, Faculty of Science and Technology, NOVA University of Lisbon, Caparica Campus, 2829-516 Caparica, Portugal; pmv@fct.unl.pt; 2 Department of Pediatrics, Hospital Garcia de Orta, EPE, 2805-267 Almada, Portugal; acosta@hgo.min-saude.pt

**Keywords:** jaundice, bilirubin, newborns, signal quality, machine learning

## Abstract

Bilirubin is a product of the metabolism of hemoglobin from red blood cells. Higher levels of bilirubin are a sign that either there is an unusual breaking down rate of red blood cells or the liver is not able to eliminate bilirubin, through bile, into the gastrointestinal tract. For adults, bilirubin is occasionally monitored through urine or invasive blood sampling, whilst all newborns are routinely monitored visually, or non-invasively with transcutaneous measurements (TcBs), due to their biological immaturity to conjugate bilirubin. Neonatal jaundice is a common condition, with higher levels of unconjugated bilirubin concentration having neurotoxic effects. Actual devices used in TcBs are focused on newborn populations, are hand-held, and, in some cases, operate in only two wavelengths, which does not necessarily guarantee reliable results over all skin tones. The same occurs with visual inspections. Based on that, a continuous bilirubin monitoring device for newborns is being developed to overcome visual inspection errors and to reduce invasive procedures. This device, operating optically with a mini-spectrometer in the visible range, is susceptible to patient movements and, consequently, to situations with a lower signal quality for reliable bilirubin concentration estimates on different types of skin. Therefore, as an intermediate development step and, based on skin spectra measurements from adults, this work addresses the device’s placement status prediction as a signal quality indication index. This was implemented by using machine learning (ML), with the best performances being achieved by support vector machine (SVM) models, based on the spectra acquired on the arm and forehead areas.

## 1. Introduction

Jaundice is an abnormal yellowing of the skin, sclerae, or mucous membranes due to the accumulation of bilirubin in these tissues [1]. According to epidemiology data, the jaundice incidence variation is dependent on the underlying cause and is more frequent in certain age groups. For example, jaundice due to alcoholic and non-alcoholic liver diseases is predominant in men, while primary biliary cholangitis as an underlying cause of jaundice is more often seen in women [2,3]. Neonatal jaundice, which is the focus of this work, occurs at rates of 50% for term newborns and 80% for preterm newborns. These significant rates and the possibility of evolution to an encephalopathy lead to a strong recommendation to routinely monitor newborns for the development of jaundice [4].

Although visual inspections are recommended as the first patient approach, they are classified as not reliable to estimate bilirubin levels in newborns; thus, bilirubin levels should be measured non-invasively by transcutaneous bilirubinometers or invasively with serum bilirubin analyses [5,6,7]. The measurement of the total serum bilirubin (TSB) is an invasive, time-consuming, and stressful procedure. Otherwise, the monitoring of the transcutaneous bilirubin (TcB) is a reliable and non-invasive method that can decrease the number of blood samples required for jaundice evaluation, or even avoid the need to collect them; however, it can still be improved with a better correlation between TSB and TcB in measurement devices [8,9].

TcB measurements are analyses of the skin’s diffuse reflectance when it is exposed to different wavelengths. The spectral content of measured light depends on the concentration of the different chromophores in the skin and subcutaneous tissues. Therefore, through absorption spectral differences, the TcB level can be calculated. Thus, beyond blood sparing, TcBs easily allow for more frequent measurements, being of great value for preterm neonates, which have more risk factors that predispose them to neurotoxicity, or critically ill babies, who have already been subjected to painful procedures [10,11].

Also, as jaundice management is carried out over time, some studies reinforce the analysis of the rate of rise in bilirubin as a predictor for risk designation, or as an indicator for phototherapy timing and duration, or even for early discharge policies in term and late preterm neonates [12,13,14,15]. Still considering the bilirubin rate of rise as a clinical feature, a study by [16] developed machine learning (ML) models to predict subsequent bilirubin measurements and provide advanced clinical decision support. Therefore, based on the above-presented scenarios, a non-invasive wearable device is being developed for continuous monitoring of bilirubin.

**Figure 1:**
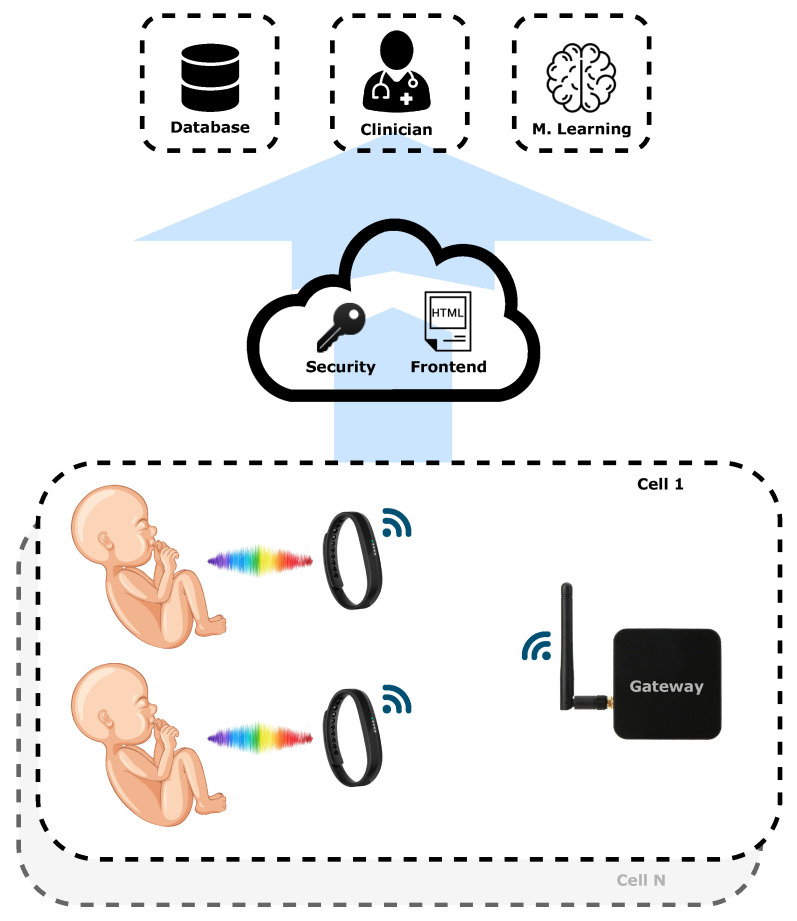
System structure.

The system’s operative environment of this wearable device could be inside a hospital or at the houses of patients. As presented in Figure 1, an example scenario could be based on measurement cells with non-invasive continuous measurement devices installed on newborn patients. Each cell has a gateway that controls and reads the acquired data of the devices via short-range wireless communication. Beyond this, each gateway communicates through a mobile or wired network to the web server, which records the data in a database, enabling the operation of ML algorithms for data classification. A clinical front-end for data visualization and alarm settings is also presented.

However, as pointed out by [17], signal quality is a common challenge in wearables. This occurs because wearables are continuous health-monitoring devices, in which the production rate of errors and false alarms, for example, can be high due to unavoidable factors, such as motion artifacts, saturation, or environment noise [18]. In the case of TcBs, these above-mentioned factors are even critical, as there is no prior knowledge of the skin tone under evaluation. Therefore, the evaluation of signal quality is crucial for achieving satisfactory results with relevant data accuracy and reliability [19,20,21], especially in the analysis of bilirubin levels with continuous TcBs. Thus, the aim of this article is to investigate the use of ML models to evaluate signal quality during continuous skin spectral acquisition. This first analysis was conducted on adults, as an intermediate validation step. In a next future step, transcutaneous bilirubin measurements will be performed in newborns and then could be integrated into hospitals’ clinical processes.

## 2. Materials and Methods

The skin has three main chromophores: melanin, hemoglobin and bilirubin. They all interact optically on the visible range but with different intensities along this spectrum region. Bilirubin absorption peak occurs around 460 nm. However, as the other two main chromophores impact the bilirubin evaluation on the skin, the reflectance analysis on this wavelength only is not enough and can be improved by adding multiple other wavelength measurements. This approach was used in this work and it is recommended for subjects with different skin colors [22,23].

More information detailed below, about the prototype and the sensor module for measurement acquisition, will guide the understanding of the test scenario. Besides that, this section explains the measurement protocol, the quality assessment and the feature engineering process performed over the acquired spectra.

### 2.1. Sensor

**Figure 2:**
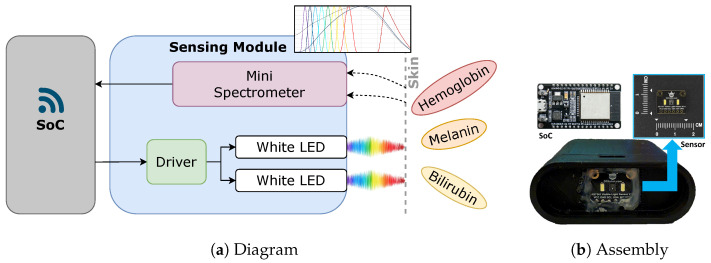
Prototype with SoC and Multi-Spectral Digital Sensor AS7341.

The sensing module uses the Multi-Spectral Digital Sensor AS7341 from ams OSRAM (Munich, Germany), the spectral response of which is defined by 8 individual channels centered in the Visible Spectrum (VIS), 415, 445, 480, 515, 555, 590, 630, 680 nm plus one extra channel at 910 nm. All channels have a resolution of 16-bits. This sensor is integrated into a 20 mm × 18 mm board and a detailed view of this design can be seen in Figure 2b. Two Surface-Mount Device (SMD) white LEDs, with their driver, work as the light source. According to [24], this sensor can detect multiple analytes by monitoring the fluorescence of quantum dots mixtures, with different colors, in concentrations as low as 0.3 nM, making it suitable for biological applications. Another study performed by [25] evidenced a significant correlation (R^2^ = 0.9999) between the measurements with AS7341 and standard table-top spectrophotometers in healthcare applications.

### 2.2. Prototype

A System-on-Chip (SoC) enables the control of the sensor through a short-range wireless network. While two white LEDs generate the light stimulus in the VIS range, the sensor AS7341 captures the reflected data samples every 2 s with an integration time of 0.5 s. This sampling value is related to the bilirubin dynamic being relatively slow, with perceptive changes in an hour time base. Besides that, being a handheld or wearable device, it is susceptible to loss of signal situations related to body movements, which means a poor optical coupling between the sensor and the skin. Therefore, to guarantee valid measurements, it is necessary to evaluate the quality of the signal as a first step analysis and then proceed with the chromophores concentration evaluation more in deep. From a mechanical perspective, the sensor stands off 10 mm from the skin and the intensities or gain are adjusted by Inter-Integrated Circuit (I2C). The diagram of the developed prototype is presented in Figure 2a and the prototype used to perform the measurements of this work can be viewed in Figure 2b.

In the actual scenario, when the device is connected, the measured data are sent through notifications to a short-range wireless communication gateway wired to a local server, in which the data are visualized, stored and processed. Otherwise, the data are discarded.

### 2.3. Measurement Protocol

**Figure 3:**
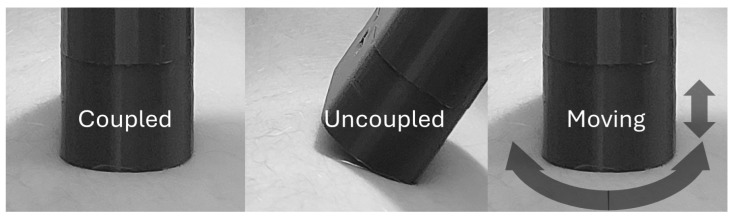
Device coupling states detailed.

The measurement sequence follows a developed protocol of 3 possible placement scenarios for wearables: coupled, uncoupled and moving, as exemplified in Table 1. By starting on a well-coupled position at the skin, the placement evolves to a smooth inclined (uncoupled) arrangement. At the end, the user performs rotational movements touching the skin or with eventual lift-offs (moving). These device placements are presented in Figure 3. Since the slow dynamics of the bilirubin concentration, a coupling perception requires more than 3 measurements. Therefore, the trials were segmented in time windows of 15 s, which resulted in around 7 samples per window. Between 2 and 4 windows are suggested for each scenario, resulting in at least 6 windows per trial. The acquisitions were performed in indoor environments with different light levels.

### 2.4. Quality Analysis

The acquired measurements were automatically windowed and manually labeled, based on the presented protocol and on the measurement signal characteristics. Two broader quality categories were defined: usable (high probability of having significant information) and not usable (low probability of having meaningful information due to artifacts or invalid samples). In the usable category, only one target is considered according to the device position: well coupled to the skin. Inside the not usable category, two possibilities were identified: uncoupled and moving.

**Figure 4:**
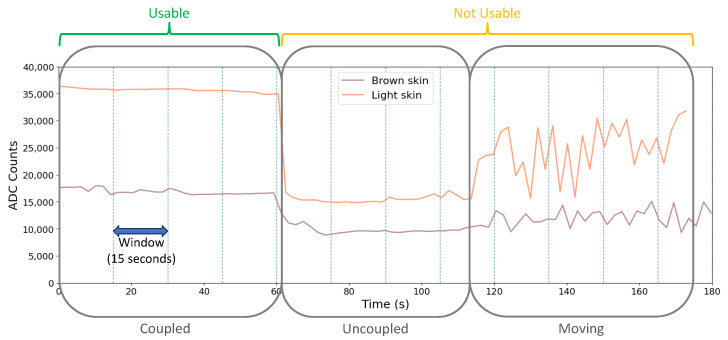
Windowing and labeling for signals from brown and light skin in the 590 nm wavelength.

Signal quality investigations performed by rule-based techniques normally use statistical features and a set of thresholds for signal classification [26]. This approach might be inaccurate for the wearables scenario due to, for example, the dynamics of the motion artifacts [27]. Also, different reflectance levels are obtained from different skin tones. On channel 590 nm (Figure 4), for example, the signal characteristics obtained from a brown skin tone arm, in a well-coupled scenario, are very similar to a light skin tone arm in an uncoupled scenario. Machine learning supervised methods can achieve better performances for these applications, by using support vector machine (SVM), Decision Trees, Random Forest, as well as unsupervised methods or, more recently, deep learning techniques. However, they present more challenges for real-time deployments, concerning computationally efficient algorithms and hardware designs [18,28]. Therefore, a machine learning-supervised approach was selected for this case, as it adequately fits the amount and type of data from the presented application. All the ML procedures were performed in the open source data mining suite [29].

A label was considered for each window. Therefore, even when evaluating just one or more channels in the respective window, the selected label is applied to all channels. An example of trial windowing and labeling is presented in Figure 4.

### 2.5. Feature Engineering

The features were selected from studies involving motion and signal quality analysis in heart rate measurement wearables. They are simple statistical signal metrics that do not require high processing time and power but are proven to be powerful for supplying information in skin reflectance applications [17,30]. Basically, they are time domain features calculated for each time-sliced window, from all the spectrometer channels. Considering the 9 channels and 5 features per channel, a total of 45 features are extracted per window. These features are detailed below:

- **Mean:** the mean value of the window;
- **Peak-to-peak:** the subtraction of the maximum and minimum measurement values of the window;
- **Standard deviation:** the indication of how much the values differ from the mean in a window;
- **Median:** the value lying at the midpoint of observed values in the window;
- **Variance:** the measure of how far apart the values are spread out in a window.

Predicting common constraints related to wearable devices, such as battery capacity and low-power networks, two approaches for feature selection were tried: a single metric from different channels or different metrics from a specific channel. The idea was to reduce the used resources and also to prevent eventual overfitting situations.

In some ML classifiers, the feature selection process is often based on the designer’s expertise [28]. During the pre assessment phase, analysis of the data representation was performed by metrics, channels and a combination of both, verifying the classification capacity of the selected group of features. The performances from this preliminary step were compared with the use of all features and the best approach was selected for evaluation of the different ML models.

## 3. Results and Discussion

This section will present the validation of the optical measurement setup. It begins by detailing the sensor characterization and data acquisition and then the feature analysis process is exposed followed by a performance assessment of the selected classification models.

### 3.1. Sensor Characterization

**Figure 5:**
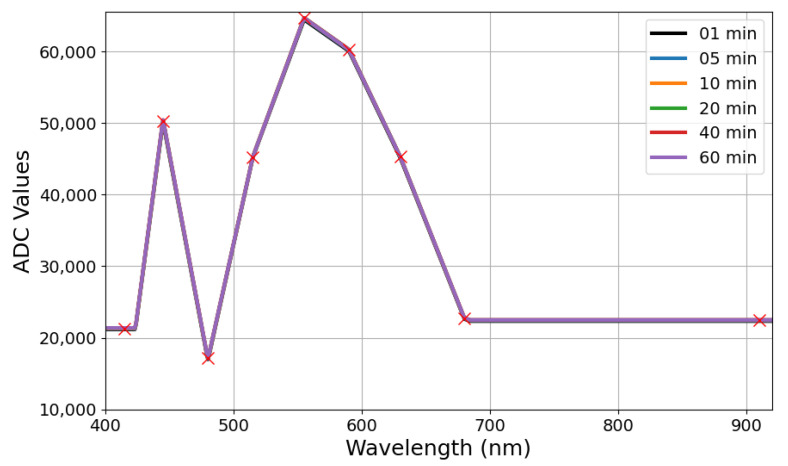
Characterization of sensor module over time.

**Figure 6:**
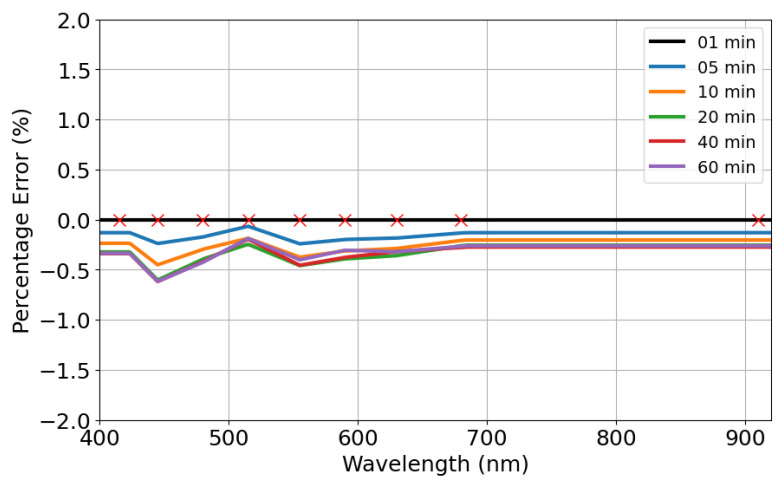
Sensor module stability error over time.

The light source spectrum characteristics were evaluated directly with the AS7341 and it reading stability was evaluated along 60 min. Basically, the prototype was placed over a white paper sheet, assuming this scenario as the maximum measured reflectance. Figure 5 shows this light source spectrum pattern acquired over time, while the measurement error, relative to the acquisition made on the first minute, is depicted in Figure 6. Markers represent the center of the micro-spectrometer channels in both figures. A peak at 450 nm and a more spread signal around the maximum level at 550 nm represent the spectrum characteristics of white LEDs. Regarding stability, the measurements revealed errors below 1% over all testing time, or below 0.5% considering a time period below 5 min.

### 3.2. Data Acquisition

This study is based on measurements performed in 10 brown and light skin tone volunteers comprising men and women with ages between 20 and 60 years old. All volunteers were informed about the system, protocol and objectives of the study. Informed consent was signed by each participant before participating and no personal data were stored. Samples were taken from the forehead and arm of each subject and the trials followed the specified measurement protocol of Table 1. As detailed in Table 2, a database with 596 windows was created. The samples were randomly segregated into 5 volunteers for training and 5 different volunteers for ML model validation, with approximately half of the windows coming from the arm and half from the forehead, also randomly selected.

**Figure 7:**
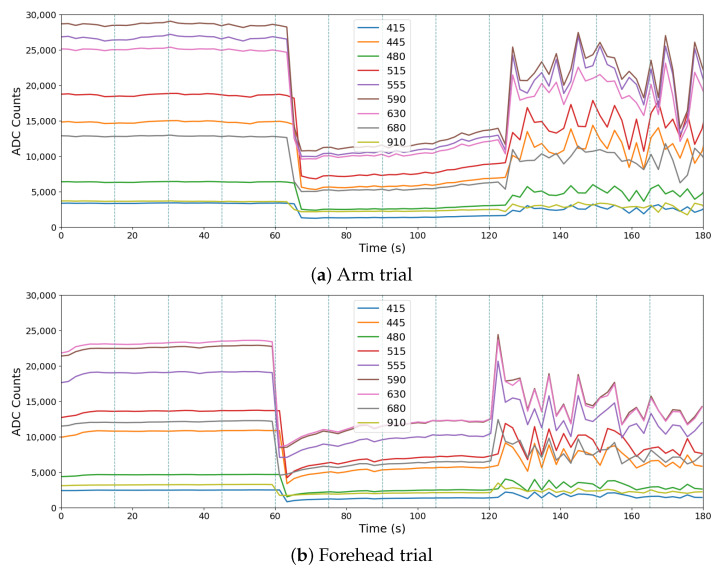
Signals from a subject in different scenarios and body placement.

A full trial measurement, with nine spectral channels, is shown in Figure 7a, from the arm, and in Figure 7b, from the forehead. They follow the suggested measurement protocol of Table 1: coupled, uncoupled and moving. In windows in which intermediate conditions occur due to transitions, the chosen label was moving. Although the different moments of the presented trials can be visually detected, it is necessary to remember that the skin tones are diverse and simple metrics evaluation can lead to mismatches in the classification process. The same mismatches can occur between measurements on different body places, as can be seen when comparing Figure 7a and Figure 7b. From that, it is possible to perceive that the relation among the channels 555, 590 and 630, for example, changes from the arm to the forehead. This happens due to small differences in the skin layer structure at those body places.

### 3.3. Feature Analysis

All the features are extracted from signal dynamic characteristics on time, called metrics, and evaluated inside each window and for all nine channels (with a total of 45 features per window). From Figure 4, it was possible to perceive that a channel’s mean is not sufficient to achieve a reliable signal quality prediction and more inputs are needed to have confident results. On the other hand, similar metrics or adjacent channels can present a high correlation among them and, eventually, a feature reduction seems feasible.

Therefore, to better understand the feature analysis performed in this section, we aggregated the features in groups. A single metric from different channels, for example, or different metrics from a specific channel. In the end, an analysis comprehending the integration of both feature groups will be exhibited. The data used in this analysis are the arm and forehead training data detailed in the previous sections.

#### 3.3.1. Metrics Analysis

To reduce the processing impact of all the channels’ metric features, we tried to evaluate the scenario’s segregation capability of one single metric extraction, but considering all the channels. The selected metric was the peak-to-peak, which has the highest score of classification accuracy in comparison to other metrics.

**Figure 8:**
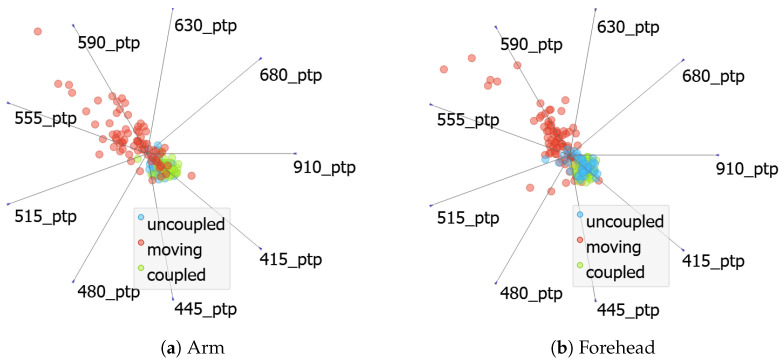
Feature analysis by metrics.

The outcomes from this analysis are based on Figure 8a,b, which represents the arm and forehead measurements, respectively. It can be perceived, in both cases, an overlapping of all scenarios, more visible in the coupled and uncoupled situations. Besides the visual evaluation, the ML model predictions were not satisfactory. Even being peak-to-peak, the indicated feature for this classification problem, other features alone were also verified, but scenarios overlapping were always present. This finding can be translated into the assumption that one metric alone is not sufficient to discriminate the target scenarios, even if it is considered over all measurement channels.

#### 3.3.2. Channel Analysis

Another perspective is the feature orientation by channel. We explored the idea of using the information from one channel, with all its metrics, to represent the required characteristics for different scenarios segregation. This approach is generally used on photoplethysmography (PPG) measurement devices based on one or two wavelengths [31]; however, the signal quality analysis is performed considering the signal shape over time and not only one sample as conducted in this work.

**Figure 9:**
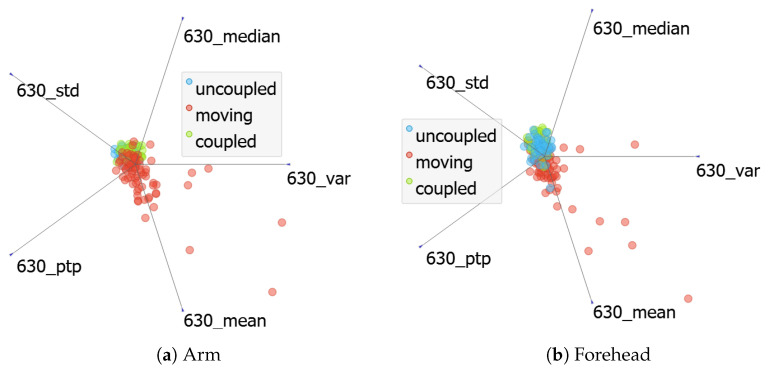
Feature analysis by channel.

The channel of 630 nm was selected for this analysis, also based on the highest classification accuracy score. Figure 9a,b present the projections for the arm and forehead, respectively. As well as in the metric analysis, the overlapping of target scenarios and poor ML model predictions also occur. Again, even with 630 nm being the most indicated channel for this classification, other channels were also evaluated individually, but overlapping scenarios were obtained. Therefore, we assume that only one channel does not concentrate all the requirements for scenarios distinguishing.

#### 3.3.3. Combined Analysis

Based on the previous section’s results, the next logic feature selection is a tentative combination of metrics and channels. By fixing the number of features at nine (to allow the selection of all the channels, if needed), the best scored features in classification accuracy were selected (Arm: 415_standard_deviation (std), 415_peak-to-peak (ptp), 445_std, 445_ptp, 480_ptp, 515_ptp, 555_ptp, 630_ptp, 680_ptp; Forehead: 415_ptp, 415_std, 445_ptp, 480_ptp, 515_ptp, 590_ptp, 630_ptp, 680_median, 680_ptp). Even in this case, the overlapping and poor predictions were evident and the graphs were similar to the projections presented previously in Figure 8 and Figure 9.

**Figure 10:**
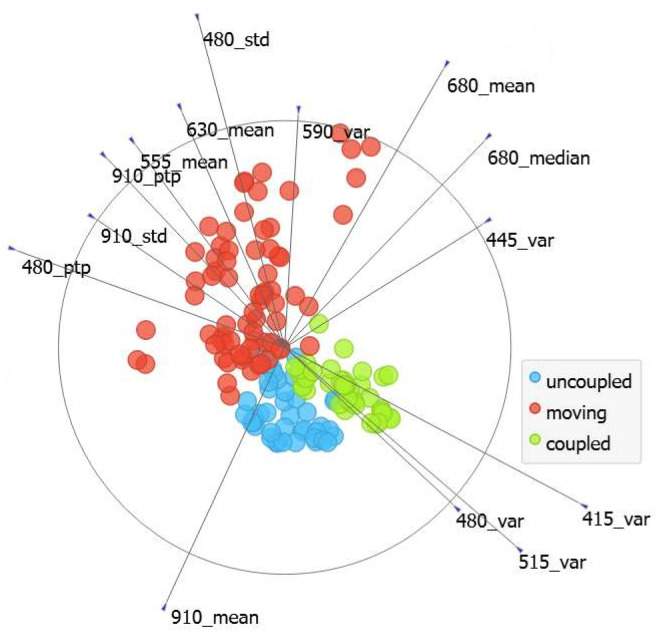
Feature visualization.

The last approach is, in fact, to use all the features. It is also important to remember that, for the final application of bilirubin measurement, all the channels will be needed to handle different skin tones. Therefore, the multivariate projection presented in Figure 10 illustrates that by using all the metrics and channels. Scenario segregation was possible in this case. This image shows linear projections with the features normalized and, although the features with longer base vector projections are more relevant for classification, all the features play a key role in the final classification capacity [32]. Inside the round circle are all the other features that contribute to this projection, but they are hidden to improve visualization. The use of all features was the selected approach for supplying the ML models that will be presented in the next section.

### 3.4. Classification Models

In the context of signal quality assessment, SVM has been used for building the signal quality classifiers [18,33]. Besides this, a comparison of Tree, Random Forest and Naive Bayes models enhances this evaluation. All the features were used for training these models using cross-validation with five folds. After the models’ development, they performed predictions over the validation data subset. The parameters of the models were fine-tuned according to the body placement location; therefore, different models were applied for the arm and forehead. The performance of the models in both cases is presented below.

In Table 3, each model’s performance over the arm is represented by the area under the receiver-operating curve (AUC), classification accuracy (CA), precision (F-score) and specificity (SPEC). For this case, the SVM model presents the highest scores for all performance statistics, confirmed by a stable high score in the confusion matrix analysis.

**Figure 11:**
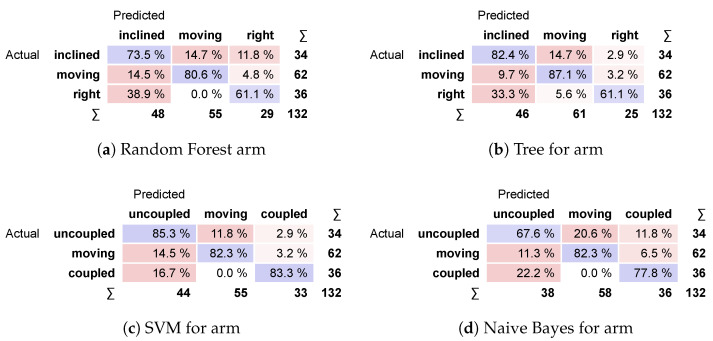
Confusion matrices for arm.

The classification performance of the models can be compared for each target in the confusion matrix, as seen in Figure 11 for the arm. It integrates the prediction percentage for each target over their true values. Even with the Tree model reaching 87% of correct predictions for moving scenarios, it falls to 61.1% for coupled scenarios. The same occurs for other models except for SVM, from which the results are more stable and all scores are equal to or higher than 82.3%.

The classification performances of the models at the forehead are in Table 4. The highest scores depend on which statistic is evaluated, but Random Forest and SVM have the best ones. Again, the SVM model was selected because it scores significantly higher than other models for the coupled scenario while keeping or increasing the score for uncoupled and moving scenarios.

**Figure 12:**
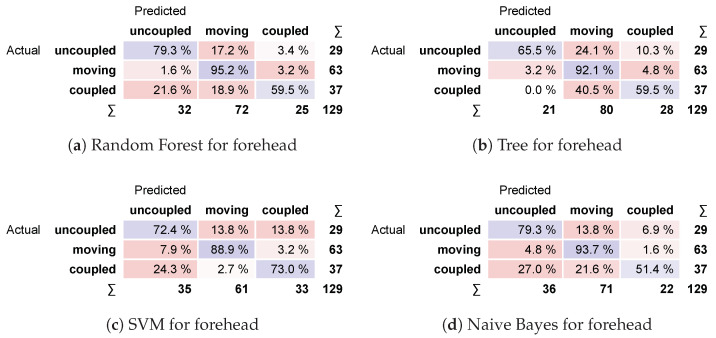
Confusion matrices for forehead.

The confusion matrix for the forehead is shown in Figure 12. Most models present highly accurate predictions for moving scenarios, reaching values equal to or above 92.1%; however, the same models score below 60% for coupled scenarios. Again, the exception is the SVM model, which reaches 88.9% for moving, while keeping a score of 73% for coupled.

Therefore, although different models are used for the arm and the forehead, SVM is the best model suggestion for both cases. By having the possibility of knowing the sensor placement a priori, the results could be impacted positively by adjusting the ML model parameters accordingly.

Besides the scoring, another comparison between models is the computational complexity, which can have a significant impact on wearable applications. Table 5 presents a simple normalized time complexity of the models under investigation. SVM presents the highest time complexity, while Tree the lowest.

## 4. Conclusions

The use of wearables for health-related measurements has been studied due to the possibility of increasing the capacity to monitor and control physical parameters, even at home [34]. Proving this device’s ability to identify the skin coupling status could enable its use from birth to the first days of the newborn life. It ensures effective bilirubin monitoring (without pain for babies) and digital data history, which could help clinicians better manage the patients under neonatal care. Future work with newborn data will validate this assumption. However, the continuous measurements provided by wearables naturally suffer from movements and environmental artifacts, which leads to inaccurate results [35]. As bilirubin dynamics are slow, that is, tens of minutes or hours, the approach of signal quality evaluation used in this study proved to be enough to guarantee necessary samples with relevant information for bilirubin concentration calculation on different skin tones.

The sensor module measurement stability is a key factor in this kind of application, and the obtained results are comparable to other studies of optical wearable devices for skin inspection [36]. In this work, AS7341 was the main sensing component, as in [37]; however, in this cited study, the device application was for non-invasive blood glucose prediction, in which SVM was also used for glucose level prediction based on all the sensor channels. Therefore, our present work, together with this and other presented references, reinforced the capacity of the AS7341 multi-spectral sensor to be used in biomedical applications.

As pointed out by [38], ML is a terrific helper for wearable systems. Indeed, this work demonstrates with scores around 80% that the ML predictions, from SVM models in this case, are reliable for identifying measurement windows with relevant spectral content. This is crucial for the process of bilirubin concentration calculus, which is strongly based on removing the influence of other chromophores (melanin and hemoglobin) by using specific wavelength relationships in the spectrum. However, as SVM presents a higher computational complexity, its impact on the final application environment must be evaluated.

The possibility of feature reduction was explored, but considering the actual universe of features, the impact on the final results did not improve significantly. However, a deeper analysis with a focus on similar metrics and adjacent channels will be conducted in the next steps, by evaluating also its impact on the skin chromophores estimation. If further improvements in scenario classification will still be required, when operating on bigger datasets, other feature reduction approaches could be considered, for example, the one presented by [39], which minimizes the bias due to correlation among features.

Future work should include the calculation of bilirubin concentration for well-coupled scenarios and explore different measurement protocols, environments, and spectrometer integration times to find the right balance between movement artifact detection and improved skin chromophores estimation. Also, the deployment of the proposed signal quality evaluation solution in real-time and autonomously could help to achieve better bilirubin concentration estimates, as the device user or responsible clinician could easily be informed about mismatches in placement and correct them if necessary. The artifact removal procedures extensively used on heart rate analysis, such as the one presented in [40], should be avoided for signal recovery from uncoupled and moving scenarios in this bilirubin-related application due to the increased possibility of mistakes regarding skin tones and difficulties in calibration, since the device does not have prior knowledge of on which type of skin the measurements will be performed. Another correction approach that can be explored, if necessary, is the implementation of signal correction algorithms at the bilirubin calculation level, being operated, for example, on an hourly basis through simple trending statistics evaluation of multiple samples of bilirubin concentration.

## Figures and Tables

**Table 1:** Measurements Protocol.

Scenario	Coupled	Uncoupled	Moving
**Time (s)**	0 to 60	60 to 120	120 to 180

**Table 2:** Data organization.

Placement	Training	Validation	Total
**Arm**	153	132	285
**Forehead**	182	129	311

**Table 3:** Models scoring for arm.

Models	AUC	CA	F1	Spec
Random Forest	0.87	0.73	0.74	0.88
Tree	0.84	0.79	0.79	0.90
SVM	0.95	0.83	0.84	0.92
Naive Bayes	0.93	0.77	0.77	0.89

**Table 4:** Model scoring for forehead.

Models	AUC	CA	F1	Spec
Random Forest	0.93	0.81	0.81	0.88
Tree	0.92	0.77	0.76	0.81
SVM	0.84	0.81	0.81	0.92
Naive Bayes	0.93	0.78	0.77	0.87

**Table 5:** Models computational complexity comparison.

Models	Normalized Time Complexity
Random Forest	0.71
Tree	0.10
SVM	1.00
Naive Bayes	0.59

## Data Availability

The data that support the findings of this study are available under a Creative Commons license from the corresponding author, F.C., upon reasonable request.

## References

[1] Jones R., Britten N., Culpepper L., Gass D.A., Grol R., Mant D., Silagy C. (2004). Oxford Text-Book of Primary Medical Care.

[2] Pavlovic M.A., Stojkovic L.M., Mijac D., Milovanovic T., Dragasevic S., Sokic Milutinovic A., Krstic M.N. (2022). Jaundice as a Diagnostic and Therapeutic Problem: A General Practitioner’s Approach. Dig. Dis..

[3] Purohit T., Cappell M.S. (2015). Primary biliary cirrhosis: Pathophysiology, clinical presentation and therapy. World J. Hepatol..

[4] World Health Organization—Effective Perinatal Care (EPC): Neonatology. https://iris.who.int/bitstream/handle/10665/108599/WHO-EURO-2010-7105-46871-68344-eng.pdf?sequence=1&isAllowed=y.

[5] World Health Organization Recommendations on Newborn Health. https://apps.who.int/iris/bitstream/handle/10665/259269/WHO-MCA-17.07-eng.pdf.

[6] Slusher T.M., Zipursky A., Bhutani V.K. (2011). A Global Need for Affordable Neonatal Jaundice Technologies. Semin. Perinatol..

[7] National Institute for Health and Care Excellence—Jaundice in Newborn Babies under 28 Days. https://www.nice.org.uk/guidance/cg98.

[8] Ercan S., Özgün G. (2018). The accuracy of transcutaneous bilirubinometer measurements to identify the hyperbilirubinemia in outpatient newborn population. Clin. Biochem..

[9] Jnah A., Newberry D.M., Eisenbeisz E. (2018). Comparison of Transcutaneous and Serum Bilirubin Measurements in Neonates 30 to 34 Weeks Gestation before, during, and after Phototherapy. Adv. Neonatal. Care.

[10] Engle W.D., Jackson G.L., Engle N.G. (2014). Transcutaneous bilirubinometry. Semin. Perinatol..

[11] Lyngsnes Randeberg L., Roll B.E., Nilsen N.L.T., Christensen T., Svaasand L.O. (2005). In vivo spectroscopy of jaundiced newborn skin reveals more than a bilirubin index. Int. J. Paediatr..

[12] Hahn S., Bührer C., Schmalisch G., Metze B., Berns M. (2019). Rate of rise of total serum bilirubin in very low birth weight preterm infants. Paediatr. Res..

[13] Thakkar P., Chavda H., Doshi V. (2017). Transcutaneous bilirubin nomogram for healthy term and late preterm neonates in first 96 hours of life. Indian Pediatr..

[14] Bhutani V.K., Johnson L., Sivieri E. (2008). Predictive Ability of a Predischarge Hour-specific Serum Bilirubin for and Near-term Newborns. Pediatrics.

[15] Ning Z., Long Z., Yang G., Xing L., Xue X. (2022). Self-Powered Wearable Biosensor in a Baby Diaper for Monitoring Neonatal Jaundice through a Hydrovoltaic-Biosensing Coupling Effect of ZnO Nanoarray. Biosensors.

[16] Chou J. (2020). Predictive Models for Neonatal Follow-Up Serum Bilirubin: Model Development and Validation. JMIR Med. Inf..

[17] Veiga P., Varandas R., Gamboa H. (2023). Machine Learning Algorithm Development and Metrics Extraction from PPG Signal for Improved Robustness in Wearables. BIOSTEC.

[18] Reddy G.N.K., Manikandan M.S., Murty N.V.L.N. (2020). On-Device Integrated PPG Quality Assessment and Sensor Disconnection/Saturation Detection System for IoT Health Monitoring. IEEE Trans. Inst. Meas..

[19] Böttcher S., Vieluf S., Bruno E., Joseph B., Epitashvili N., Biondi A., Zabler N., Glasstetter M., Dümpelmann M., Van Laerhoven K. (2022). Data quality evaluation in wearable. Sci. Rep..

[20] Bent B., Goldstein B.A., Kibbe W.A., Dunn J.P. (2020). Investigating sources of inaccuracy in wearable optical heart rate sensors. NPJ Dig. Med..

[21] Goldsack J.C., Coravos A., Bakker J.P., Bent B., Dowling A.V., Fitzer-Attas C., Godfrey A., Godino J.G., Gujar N., Izmailova E. (2020). Verification, analytical validation, and clinical validation (V3): The foundation of determining fit-for-purpose for Biometric Monitoring Technologies (BioMeTs). NPJ Dig. Med..

[22] Yan L., Hu S., Alzahrani A., Alharbi S., Blanos P. (2017). A Multi-Wavelength Opto-Electronic Patch Sensor to Effectively Detect Physiological Changes against Human Skin Types. Biosensors.

[23] Chang C., Wu C., Choi B., Fang T. (2019). MW-PPG Sensor: An on-Chip Spectrometer Approach. Sensors.

[24] Huerta J., Pirbhai M. (2023). Exploring the Potential of a Multispectral-Sensing System with Automated Machine Learning for Multiplex Detection. IEEE Sen. J..

[25] Jesuraj A., Hassan U. (2023). Point-of-Care Portable 3D-Printed Multispectral Sensor for Real-Time Enzyme Activity Monitoring in Healthcare Applications. Biosensors.

[26] Vadrevu S., Manikandan M. (2019). Real-Time PPG Signal Quality Assessment System for Improving Battery Life and False Alarms. IEEE Trans. Circ. Syst. II.

[27] Mahmoudzadeh A., Azimi I., Rahmani A., Liljeberg P. (2021). Lightweight photoplethysmography quality assessment for real-time IoT-based health monitoring using unsupervised anomaly detection. Proc. Comp. Sci..

[28] Seng K., Ang L., Peter E., Mmonyi A. (2023). Machine Learning and AI Technologies for Smart Wearables. Electronics.

[29] Orange Data Mining. https://orangedatamining.com/.

[30] Cosoli G., Antognoli L., Scalise L. (2023). Wearable Electrocardiography for Physical Activity Monitoring: Definition of Validation Protocol and Automatic Classification. Biosensors.

[31] Moscato S., Giudice S., Massaro G., Chiari L. (2022). Wrist Photoplethysmography Signal Quality Assessment for Reliable Heart Rate Estimate and Morphological Analysis. Sensors.

[32] Demšar J., Leban G., Zupan B. (2007). FreeViz-An intelligent multivariate visualization approach to explorative analysis of biomedical data. J. Biom. Inf..

[33] Orphanidou C., Drobnjak I. (2017). Quality assessment of ambulatory ECG using wavelet entropy of the HRV. IEEE J. Biomed. Health Inf..

[34] Ma X.L., Chen Z., Zhu J.J., Shen X.X., Wu M.Y., Shi L.P., Du L.Z., Fu J.F., Shu Q. (2020). Management strategies of neonatal jaundice during the coronavirus disease 2019 outbreak. World J. Pediatr..

[35] Inamori G., Kamoto U., Nakamura F., Isoda Y., Uozumi A., Matsuda R., Shimamura M., Okubo Y., Ito S., Ota H. (2021). Neonatal wearable device for colorimetry-based real-time detection of jaundice with simultaneous sensing of vitals. Sci. Adv..

[36] Cusini I., Rinaldi R., Castiglioni P., Faini A., Villa F. (2023). Multi-wavelength SPAD photoplethysmography for cardio-respiratory monitoring. Front. Phys..

[37] Pham Q.H., Nguyen T.N., Ba A.Q.H., Ngo H.H., Vo H.H., Tran N.T. An Embedded System for Non-Invasive Glucose Monitoring. Proceedings of the International Conference on Multimedia Analysis and Pattern Recognition (MAPR).

[38] Deng Z., Guo L., Chen X., Wu W. (2023). Smart Wearable Systems for Health Monitoring. Sensors.

[39] Xue B., Shi W., Chotirmall S.H., Koh V.C.A., Ang Y.Y., Tan R.X., Ser W. (2022). Distance-Based Detection of Cough, Wheeze, and Breath Sounds on Wearable Devices. Sensors.

[40] Xia S., Wung S.F., Chen C.C., Coompson J.L.K., Roveda J., Liu J. (2024). Data-Fusion-Based Quality Enhancement for HR Measurements Collected by Wearable Sensors. Sensors.

